# The Abuses of Medical Charity

**Published:** 1880-04

**Authors:** 


### THE ABUSES OF MEDICAL CHARITY.

It is hardly necessary to excuse the interest which is now being taken in the question of the abuse of medical charity. In fact, in view of the enormity of the evil, and of its widespread character, it is a wonder that it claims such a comparatively small amount of the time and attention of medical men.

Possible reasons for this are, that the older men in the profession are, pecuniarily speaking, beyond the reach of the bad influences of the system, and the younger men are apt to look upon the present condition of things as inevitable, and past remedy. As a marked evidence for reform, however, it is gratifying to notice that the sentiment of the younger men is beginning to change, and in various sections of the country the abuse of medical charity is being discussed in a way that promises some practical result. The matter of making a living by his profession appeals to him with terrible earnestness. Year by year, with the increased competition going on around him, he finds that his chance for success is becoming less and less. He cannot prevent the new men from dividing up his living, and he is consequently compelled to enlarge the field of his struggle, and to look after what was heretofore considered the waste places. The idea of throwing away services for the sake of being called charitable, of struggling the best years of one's life for a bare subsistence, and, in the end, leaving a family dependent upon some widow and orphan society, is beginning to lose its romance. The necessities of the hour look beyond this. There is nothing that gives a man a better chance in a race for bread than to know that his breathing is not impeded by a full stomach. It is hardly complimentary to the younger practitioner to liken him to such a bread-winner, but it is necessary to do so in the cause of truth. Hence, we can appreciate the earnestness of his efforts for reform.

We agree with him, and with all others who have given attention to the subject, in saying that the abuse of medical charity is beyond all reason, and that the most radical measures are called for. The evil, far from abating, is on the increase. Dr. Dana, in a very suggestive and instructive paper on this subject in the present issue, gives some interesting statistics concerning this point. The ratio of increase of medical paupers in Birmingham, compared with the general increase of population of that city, is a striking fact, and will doubtless find its parallel in large cities in this country. In this city there is such a premium on medical pauperism that the latter is becoming respectable. It is considered a smart thing to dodge into the college clinic, into the dispensary, or out-patient department of a hospital, and get the opinion of a "head doctor" for nothing, and this from people who can afford to pay. The story of the dispensary patient who complained that she was kept waiting while she was paying a dollar an hour for her carriage at the door, has its counterpart in the experience of every dispensary physician and clinical attendant. We have in mind the landlord of a doctor who called upon the latter for advice at a dispensary, and when the service was refused, said he never paid for what he could get for nothing. But it is useless to multiply examples, or to prove a point already well established. The time has evidently come for the profession to do something to protect itself, and we are pleased to learn that the task is being undertaken with a freshened vigor, which promises tangible results. In Chicago and in Philadelphia large and earnest meetings of medical men discussed the subject in all its varied phases, and committees have been appointed to draw up a plan of action which shall tend to reconcile the conflicting interests of the colleges which must have clinical material, and the struggling medical men who must have practice. As in other cities, Chicago and Philadelphia are overrun with medical charities. In this city particularly, if the facilities for dispensary treatment, for hospital accommodation, and for clinical demonstration, are not curtailed, the practitioners who attend the middle and lower classes, and who are content with ordinary fees, will be left entirely out of the race.

Various plans have been suggested to remedy the evils. The payment of small fees has been tried with some benefit, but this is open to the objection that the people will get into the habit of supposing that the services are worth no more than the normal charges made. The provident plan has been discussed with much earnestness by the medical profession in Great Britain, but the practical results thus far have not been as satisfactory as was anticipated. In this country the idea of the establishment of similar institutions is beginning to assume a tangible form, and will doubtless ere long be an accomplished fact. But before the latter can be realized, much has to be done in reforming the present free dispensary system and in correcting the abuses of the college clinic. How this may be accomplished will be the subject of future discussion in these columns.—*The Medical Record.*

